# Sinococuline, a bioactive compound of *Cocculus hirsutus* has potent anti-dengue activity

**DOI:** 10.1038/s41598-023-27927-3

**Published:** 2023-01-19

**Authors:** Rahul Shukla, Richa Ahuja, Hemalatha Beesetti, Amit Garg, Charu Aggarwal, Shivam Chaturvedi, Kaushal Nayyar, Upasana Arora, Altaf A. Lal, Navin Khanna

**Affiliations:** 1 Translational Health, Molecular Medicine Division, International Centre for Genetic Engineering and Biotechnology, New Delhi, India; 2 Division of Virus Research and Therapeutics, CSIR-Central Drug Research Institute, Lucknow, India; 3 Sun Pharmaceutical Industries Limited, Gurugram, India; 4 Translational Health Science and Technology Institute, NCR Biotech Science Cluster, Faridabad, India; 5 Present Address: Virology Division, Foundation for Neglected Disease Research, 20A, KIADB Industrial Area Veerapura, Doddaballapur, Bengaluru, Karnataka 561203 India

**Keywords:** Drug discovery, Microbiology

## Abstract

Dengue virus (DENV) infection has increased worldwide, with over 400 million infections annually, and has become a serious public health concern. Several drug candidates, new and repurposed, have failed to meet the primary efficacy endpoints. We have recently shown that Aqueous Extract of the stem of *Cocculus hirsutus* (AQCH) was effective in vitro and in vivo against DENV and was safe in humans. We now report that an active ingredient of AQCH, Sinococuline, protects against the antibody-mediated secondary-DENV infection in the AG129 mouse model. DENV infection markers were assessed, viz*.* serum viremia and vital organs pathologies-viral load, proinflammatory cytokines and intestinal vascular leakage. The treatment with Sinococuline at 2.0 mg/kg/day; BID (twice a day), was the most effective in protecting the severely DENV-infected AG129 mice. Also, this dose effectively reduced serum viremia and tissue-viral load and inhibited the elevated expression levels of proinflammatory cytokines (TNF-α and IL-6) in several vital organs. Based on these findings, it could be explored further for pre-clinical and clinical developments for the treatment of dengue.

## Introduction

Dengue is an arboviral disease caused by the dengue virus (DENV). DENV is a positive-sense RNA flavivirus with four antigenically different serotypes (DENV-1, -2, -3 and -4)^1^. Dengue infections are classified as primary or secondary dengue^2^. Primary dengue is caused by any one of the DENV serotypes to the naïve individual through the bite of an infected *Aedes* mosquito^2^. It is generally characterized as a self-limiting febrile illness with severe headache, pain behind the eyes, muscle and joint pains, nausea, vomiting and rash. A heterologous serotype causes secondary dengue after the first DENV infection^3^. Secondary dengue is often more severe and potentially fatal. Patients experience severe abdominal pain, persistent vomiting, plasma leaking, fluid accumulation, respiratory distress, restlessness and organ impairments^4–6^. The prevalence of all four DENV serotypes poses a significant risk of secondary dengue infections due to antibody-dependent enhancement (ADE)^7,8^. The ADE phenomenon could convert mild dengue fever (DF) into severe dengue disease, which potentially triggers dengue haemorrhagic fever (DHF), dengue shock syndrome (DSS) and death^9,10^.

Increased travel and urbanization have increased dengue cases worldwide^11,12^ and is estimated to cause ~ 400 million DENV infections annually, of which ~ 100 million cases manifest clinical symptoms^13^. Asia alone accounts for ~ 70% of the global dengue burden while India's contribution stands at ~ 34%, making it hyper-endemic for dengue^13^. The economic burden due to DENV infection has increased and is estimated to be ~ 8.9 billion US dollars per 2013 price index^14^. Ineffective vector control measures and global warming further aggravate the dengue situation^15,16^. The investigational drugs tested so far have not shown to be effective against dengue infection^17–19^. Several repurposed drugs like: balapiravir^20^, celgosivir^21^, chloroquine^22^, lovastatine^23^ and prednisolone^24^, have failed to show clinical efficacy against dengue virus infection. These drugs could not meet primary clinical endpoints, including serum viremia and serum NS1 antigen reduction. Recently, the FDA-approved anthelmintic drug, Niclosamide was found to be effective against DENV, WNV, JEV, YFV and ZIKV in vitro^25–28^. It reduces viral replication and increases the survival but cannot completely protect DENV challenged mice^25^.

In our previous study, we showed that the aqueous extract of the stem of *Cocculus hirsutus* (AQCH) was efficacious in vitro and in vivo against DENV infection^29^. AQCH protected the severely DENV-infected AG129 mice by reducing the serum viremia and tissue-viral load. Additionally, AQCH reduced intestinal blood vascular leak and proinflammatory cytokines in these mice. Further, out of the five chemical compounds identified from AQCH, only Sinococuline was effective against all four serotypes of DENV in an in vitro virus-inhibition assay^29^.

The current study aims to delineate Sinococuline’s anti-DENV inhibitory property by evaluating its protective efficacy in the secondary dengue AG129 mouse model. Sinococuline was much more effective when administered intraperitoneally (IP) over the oral gavage in severely DENV-infected AG129 mice. The IP injection of Sinococuline (BID) adequately suppressed the intestinal vascular leakage of infected mice. It also effectively reduced serum viremia and tissue-viral load and fully protected the AG129 mice against secondary DENV infection. Importantly, Sinococuline significantly decreases proinflammatory cytokines in different organs without any adverse effect, when evaluated through a liver function test. The liver pathological investigation indicated that the explored dose of Sinococuline is very safe. Overall, these results encourage its advanced development, which will address the urgent need for an effective antiviral drug to treat DENV infection.

## Results

### Sinococuline inhibits secretion of viral antigen NS1 and prevents pan-DENV infection

**Figure 1:**
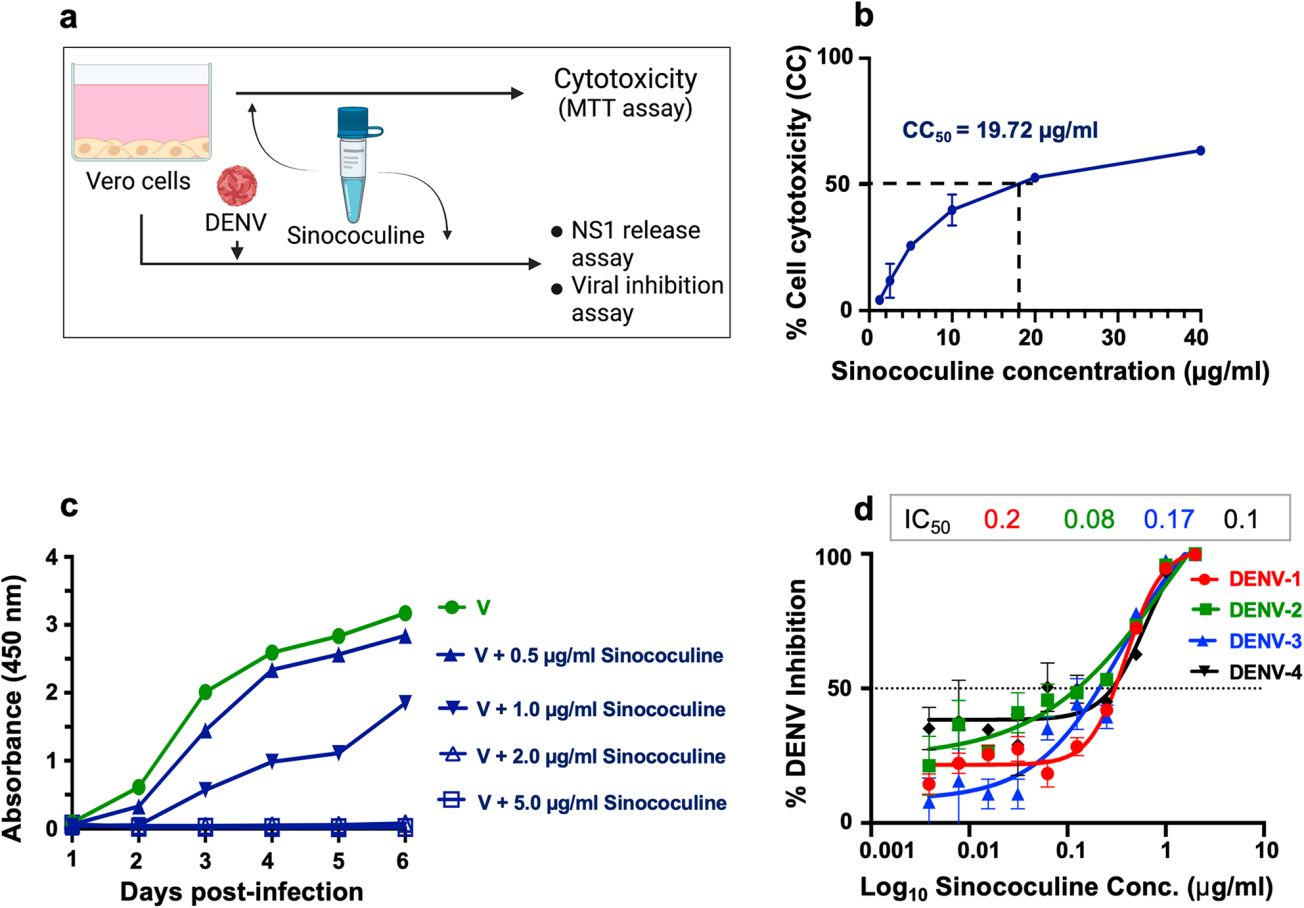
Cytotoxic effect of Sinococuline and inhibition of DENV by Sinococuline treatment. (**a**) Schematic representation of the overall protocol adapted for evaluation of cell cytotoxicity (CC) and DENV inhibition assays. (**b**) Cytotoxicity was evaluated after 48 h post infection by doing MTT assay in Vero cells and plotted the graph using GraphPad prism and the % CC_50_ was calculated. (**c**) The DENV antigen NS1 release inhibition was performed, where Vero cells were infected with DENV-2; post infection period, infection media was removed and fresh media was added. Aliquots were collected every 24 h for 6 days and NS1 ELISA was performed. Graph was plotted with days post-infection on X-axis and absorbance unit at 450 nm on Y-axis. (**d**) To calculate the inhibitory concentration of Sinococuline, Flow-cytometry-based virus inhibition assay was performed as reported previously^29^. The % DENV inhibition by Sinococuline treatment with respect to virus control (cells were infected but not treated with Sinococuline) were calculated against all four serotypes and their respective half maximal inhibitory concentration (IC_50_) was estimated by using Graphpad prism as shown in the table above to the graph. Red, green, blue and black curves represent DENV-1, -2, -3 and -4, respectively. X-axis and Y-axis denote Sinococuline concentrations in their log values and % DENV inhibition, respectively.

Mammalian cells release NS1 in the culture supernatant upon DENV infection^30,31^. This allowed evaluation of virus inhibition property of Sinococuline by analysing the inhibition of NS1 release from DENV-infected cells upon Sinococuline treatment. The assay was designed to evaluate the released NS1 amount when DENV-2 infected Vero cells were treated with different doses of Sinococuline (Fig. 1a). Based on the previous Sinococuline activity profile as reported in Shukla et al.^29^ and cytotoxicity data as shown in Fig. 1b, we selected four doses of Sinococuline (0.5 µg/ml, 1.0 µg/ml, 2.0 µg/ml and 5.0 µg/ml), for testing its efficacy in inhibiting the NS1 release by DENV infected Vero cells^29^. Aliquots of culture supernatant were collected from the treated cell culture at every 24 h intervals for six days post-infection. NS1 antigen levels were detected through a commercially available dengue NS1 antigen sandwich ELISA kit. Interestingly, we observed that Sinococuline lowered the NS1 secretion in a dose-dependent manner. In the control experiment wherein, the infected cells were not exposed to Sinococuline, dengue NS1 levels increased gradually and reached the plateau on day 4. Conversely, the lowest concentration of Sinococuline (0.5 µg/ml) decreased the NS1 antigen levels to some extent, when compared with virus control, though the reduction was statistically insignificant. However, 1.0 µg/ml of Sinococuline reduced NS1 levels significantly after day 3 (*p* = 0.023) (Fig. 1c). Notably, the 2.0 µg/ml and highest concentration (5.0 µg/ml) of Sinococuline dropped the NS1 levels significantly which was sustained over the entire period of the experiment, when correlated with virus control (*p* < 0.001). To confirm our previous report on Sinococuline anti-dengue inhibitory property, we purified Sinococuline from different batches of AQCH and assayed for pan-DENV flow cytometry-based virus inhibition assay (Fig. 1d). Expectedly, Sinococuline effectively inhibited pan-DENV infection in Vero cells; the IC_50_ of Sinococuline against each DENV was comparable with previously reported data^29^. It suggests consistency of Sinococuline in exhibiting anti-dengue inhibitory activity across the batches. The collective data suggests that Sinococuline is not toxic (CC_50_ = 19.72 µg/ml) in Vero cells and possesses pan-DENV inhibitory property with the broad selectivity index (SI) against each DENV serotypes (DENV-1: 98.6; DENV-2: 246.5; DENV-3: 116 and DENV-4: 197.2). Also, it directly affects NS1 synthesis which could be correlated with the inhibition of virus secretion in real scenarios^32,33^.

### Sinococuline is more effective through intraperitoneal route than its oral dosing in severely DENV-infected AG129 mice

In order to explore therapeutic feasibility of Sinococuline, firstly, we determined appropriate route of its administration for observing the maximum effect of Sinococuline in vivo. We selected a dose of Sinococuline (10 mg/kg/day), and administered through intraperitoneal (i.p.) injection and oral gauging in severely DENV-infected immunocompromised AG129 mice. Severe dengue infection is potentially fatal and it is mainly reported in the case of secondary DENV-infection due to ADE. To establish severe DENV-infection, 6–8-week-old AG129 mice were intravenously inoculated with immune complexes (IC) of DENV-2 strain S221 and 4G2 monoclonal antibody (mAb), as described^29,34^. Briefly, anti-flavivirus monoclonal 4G2 mAb (10 µg) was mixed with sublethal dose of mouse adapted DENV-2 strain S221 (2.0 × 10^4^ FIU) and incubated at 4 °C for 1 h. The neutralizing potency of ICs was confirmed through in vitro flow cytometry-based virus infection assay using Vero cells. Fully neutralised IC (i.e., no virus infectivity in vitro) was utilised for establishing severe DENV infection in AG129 mice which killed the mice in 4–6 days without any drug intervention, as reported previously^29^.

**Figure 2:**
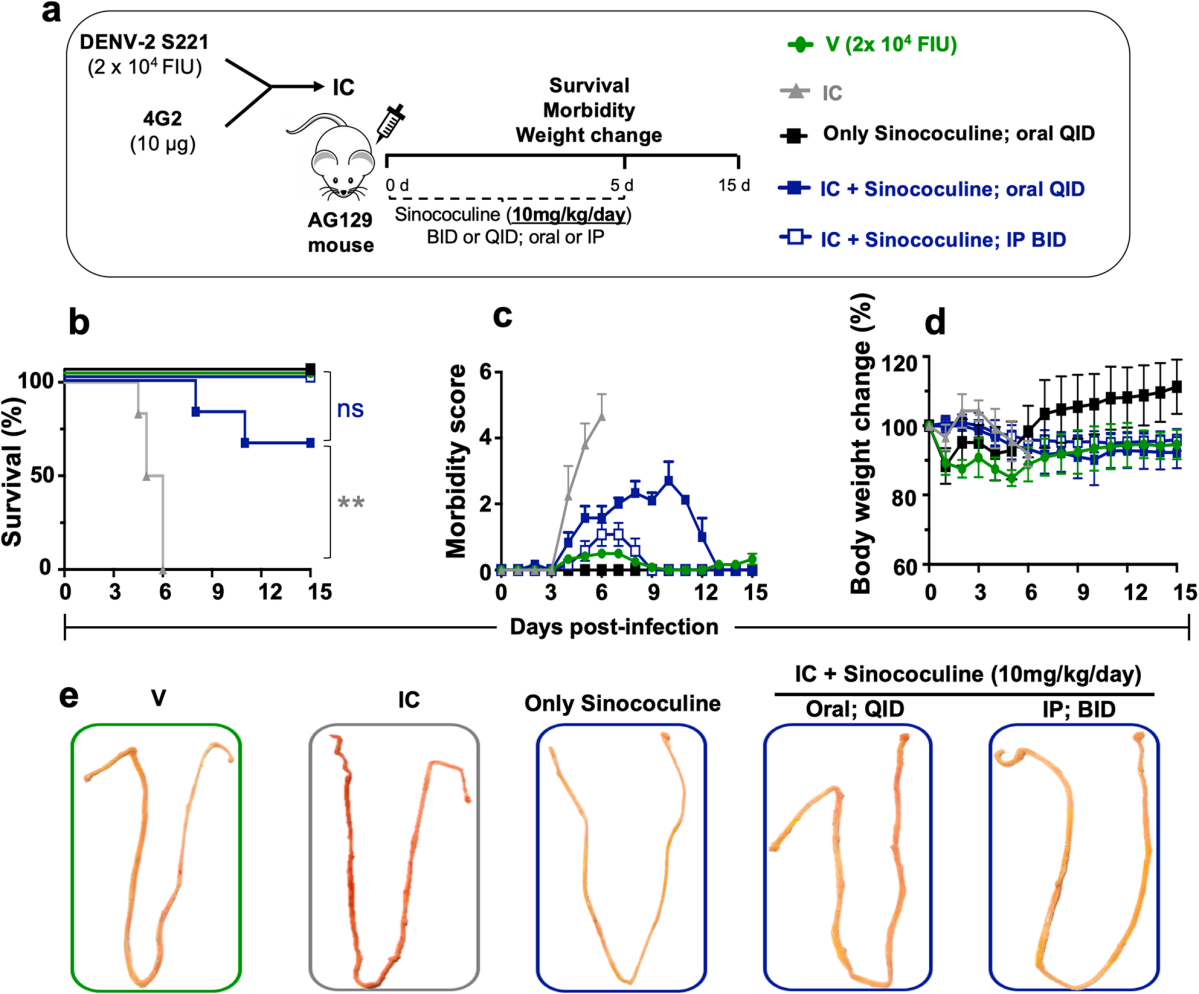
Intraperitoneal injection of Sinococuline is more effective than its oral feeding in protecting the severely DENV infected AG129 mouse model. The in vitro made IC of mouse-adapted DENV-2 S221 strain and 4G2 mAb was inoculated in AG129 mice to establish the severe DENV infection, as reported previously^29^ and Sinococuline was administered through the indicated route for evaluation of its efficacy as shown in pictorial diagram (**a**). The IC inoculated AG129 mice (n = 9) were either fed orally (four-times a day; QID; blue coloured curve with filled blue square) or intraperitoneally injected (two-times a day; BID, blue curve with empty blue square) with 10 mg/kg/day Sinococuline (made in sterile 1x PBS) for 5 days. All the mice were scored for their survival (**b**), morbidity (**c**), and body weight change (**d**) for 15 days post-infection. The only Sinococuline group was not infected with IC but fed orally with 10 mg/kg/day (QID; black curve with filled black square), considered as uninfected group. However, V: virus control mice group (green curve with filled green circle, infected with sublethal dose of DENV-2 S221, 2.0 × 10^4^ FIU/mouse) and IC group (grey curve with filled grey triangle, infected with IC) were not treated with Sinococuline and instead fed with only sterile 1× PBS (vehicle/placebo) orally (QID) for 5 days, and scored their survival, morbidity and body weight change for 15 days, similarly. The survival data (panel ‘**b**’) were analysed by Mantel–Cox test for significant difference in their survival rates, *ns* not significant, **statistically significant (*p* = 0.0010). On day 4 post-infection, a subset of mice from each group (n = 3) was euthanised, perfused with 1× PBS and their small intestinal lumens were flushed again with PBS and vascular leak was visualised qualitatively (**e**). In panel (**e**), small intestine of only one mouse from each group is represented here.

The severely DENV-infected AG129 mice (n = 9) were treated with Sinococuline (10 mg/kg/day) either via oral gauging (10 mg/kg/day; QID) or i.p injection (10 mg/kg/day; BID) (Fig. 2a). Interestingly, the IC infected mice when treated with i.p. injection of Sinococuline barely developed clinical symptoms and all mice survived over the experiment (Fig. 2b). However, mice treated orally exhibited significantly higher morbidity, reduced initial body weight (Fig. 2c,d; supplementary media file) and failed to confer complete protection (*p* = 0.1385) (Fig. 2b), despite being treated four-times a day (QID). Nonetheless, the protection of orally treated mice group was statistically significant when compared with untreated IC-inoculated group (*p* = 0.001) (Fig. 2b).

Also, we were interested in investigating the reason for high morbidity in those mice treated orally four-times a day (QID) with Sinococuline (10 mg/kg/day). A subset (n = 3) of experimental groups (described above), was euthanised on day 4 post-infection, perfused and luminal content was flushed with 1X PBS. The intestinal lumens were compared qualitatively for the evaluation of vascular leak (Fig. 2e), as the intestinal vascular leakage is one of the prominent manifestations of severe DENV infection. Notably, the intestinal lumen of mice treated orally with Sinococuline appeared to have more reddish fluid accumulation, similar to the intestine of virus control mice group. Whereas, the intestine of infected mice receiving i.p injections of Sinococuline, appeared to be normal and similar to the only Sinococuline group (sham group who received only Sinococuline; 10 mg/kg/day; BID). The collective data of survival, morbidity and appearance of small intestinal vascular leakage suggested that the Sinococuline administration via i.p injection is more effective in protecting severely DENV infected mice over oral gauging. Hence, for all our further protection studies, we administered Sinococuline through i.p injection.

### Evaluation of protective efficacy of Sinococuline at different doses in severely infected AG129 mice using the optimal route of administration

**Figure 3:**
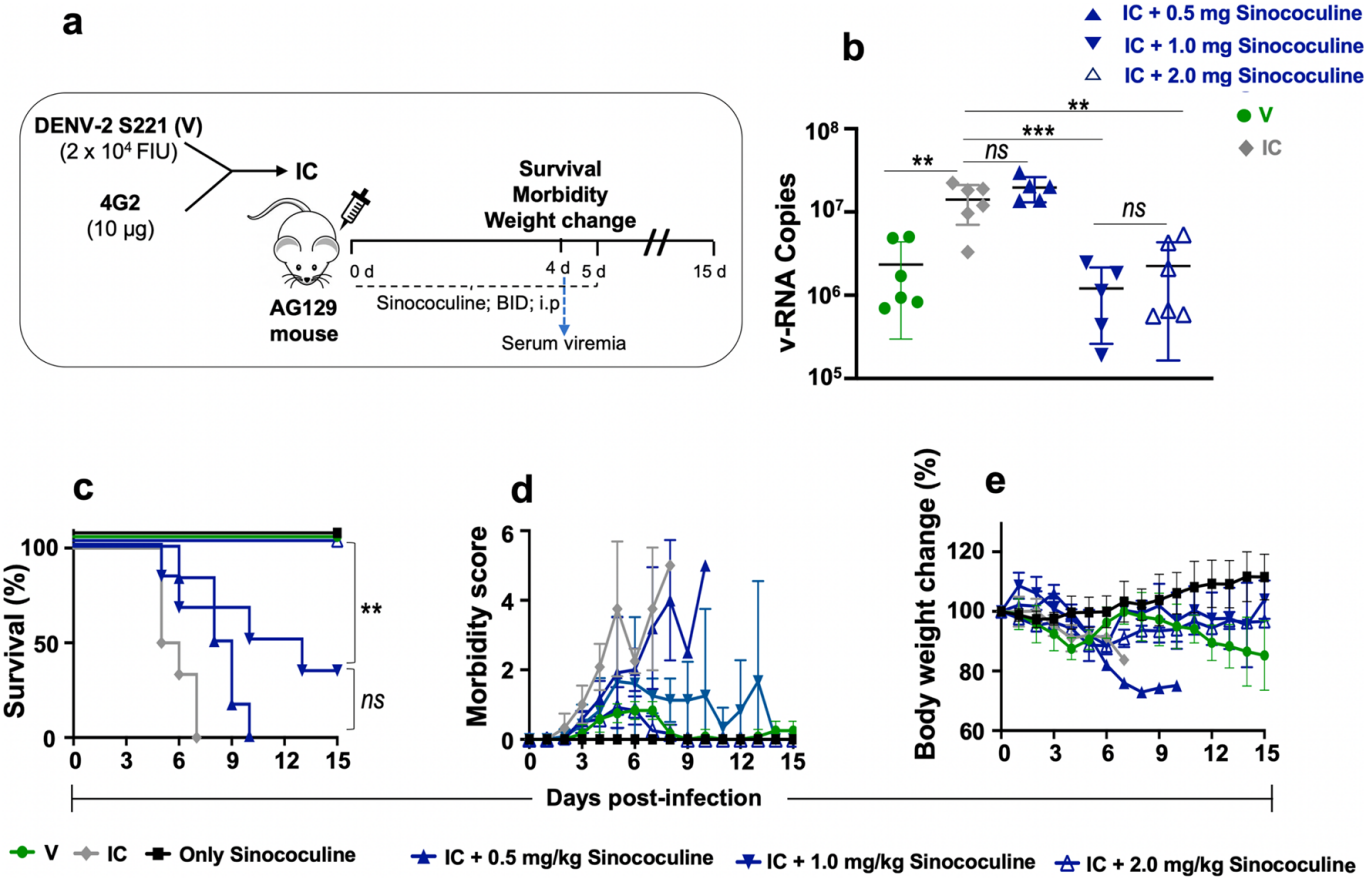
Evaluation of protective efficacy of Sinococuline in severely DENV-infected AG129 mouse model. (**a**) Pictorial representation of study design. Group of AG129 mice (n = 6) were infected with Immune complex (IC) of DENV-2 S221 and 4G2 mAb, thereafter, mice were intraperitonially (i.p) injected with three doses of Sinococuline viz*.,* 0.5 mg/kg/day (solid blue upward triangle), 1.0 mg/kg/day (solid blue downward triangle) and 2.0 mg/kg/day (empty blue upward triangle) in a twice a day (BID) dosing regimen for 5 days, and all mice were monitored for 15 days post-infection for their survival (**c**), morbidity (**d**) and body weight change (**e**). On day 4 post-infection, 50 µl of bleed were collected from each mouse for the estimation of serum viremia by performing the RT-qPCR (**b**). Control groups across all the panels were: V, mice infected with sublethal dose of virus (green circle); IC, mice inoculated with immune complex (grey diamond); only Sinococuline, the mice who were neither infected with V nor IC but injected with highest dose of Sinococuline (2.0 mg/kg/day; solid black square). To nurse all the mice similarly, both V and IC (vehicle/placebo) groups were administered i.p with 1x PBS, BID instead of Sinococuline for 5 days. Serum viremia data (panel ‘**b**’) was statistically evaluated by using Two-way ANOVA with Tukey’s Multiple comparisons test, where, *ns*: not significant (p > 0.05); ***very significant (*p* = 0.0008), **significant (*p* = 0.0013), represented on top of the data, denote statistical differences between different treated and non-treated groups. However, survival data (panel ‘**c**’) was analysed by Long-rank (Mantel–Cox) test, where *ns*: not significant (*p* > 0.05); **statistically significance (*p* = 0.0183), represented on right side of the survival graph, indicate statistical differences between the doses.

In order to evaluate optimum protective dose of Sinococuline, we utilised above described severely DENV-infected AG129 mouse model. The IC inoculated AG129 mice (n = 6) were treated with different doses of Sinococuline (0.5 mg/kg/day, 1.0 mg/kg/day and 2.0 mg/kg/day) through intraperitoneal route in a BID regimen for a period of 5 days post-infection. All assayed mice were scored for survival, morbidity and body weight change for another two weeks post initiation of treatment as shown in Fig. 3a. Notably, Sinococuline-treated groups were statistically significant than the control IC group (who did not receive any dose of Sinococuline) that developed higher morbidity scores and succumbed to the death by six days (Fig. 3c). Although, the mice treated with lowest dose of Sinococuline (0.5 mg/kg/day; BID) showed delayed mortality and survived significantly (*p* = 0.0049), but none of the mice could survive till the end of experiment (blue curve with upward triangle Fig. 3c). However, the infected mice when treated with highest concentration of Sinococuline (2.0 mg/kg/day; BID; blue curve with empty triangle) completely survived through the entire course of experiment (100% survival; Fig. 3c). Interestingly, these mice (treated with 2.0 mg/kg/day of Sinococuline) scored minimal morbidity (Fig. 3d) than other treated groups. Also, their body weight did not change significantly during the entire period of the experiment when compared with only Sinococuline administered group (black curve; Fig. 3e).

The antibody-mediated enhanced virus replication is the primary cause of severe DENV-infection. To evaluate the Sinococuline efficacy in reducing the serum viremia, the above assayed mice in Fig. 3a, were bled on day-4 for determination of viral load in the sera. The 50 µl/mouse serum was utilised for SYBR Green-based RT-qPCR assay using DENV specific primers, as reported elsewhere^29,35^. Remarkably, the IC infected mice when treated with both 1.0 mg/kg/day (downward blue filled triangle) and 2.0 mg/kg/day (upward blue empty triangle), lowered the serum viremia significantly (Fig. 3b; represented as viral copies/µl of serum) when compared with untreated or mice treated with lowest dose of Sinococuline (0.5 mg/kg/day; BID; upward blue filled triangle) (*p* = 0.0013). However, the reduction of serum viremia by 1.0 mg/kg/day Sinococuline treatment was insignificant when compared with mice treated with 2.0 mg/kg/day of Sinococuline. These results indicate that although 1.0 mg/kg/day of Sinococuline treatment was effective in lowering serum viremia, it is not sufficient to protect 100% of mice from succumbing to DENV infection.

To test the any adverse effect of Sinococuline treatment in infected AG129 mice, blood (50 µl/mouse) was collected separately on day 4 along with serum collected for the estimation of serum viremia. The collected blood was subjected for the estimation of liver function test by evaluating the essential liver enzymes available in blood samples i.e., SGOT (Serum glutamic-oxaloacetic transaminase), and SGPT (Serum glutamic pyruvic transaminase). The results indicated that Sinococuline treatment does not adversely affect the liver function in infected AG129 mice (Supplementary Fig. 1). On the contrary, the highest concentration of Sinococuline (2.0 mg/kg/day) treatment promptly reduced SGOT level, which was elevated due to severe DENV-infection (*p* = 0.0059). It is well proven that severe DENV-infection causes hepatic dysfunction, as manifested by abnormal liver enzyme levels^36–38^.

### Sinococuline treatment is effective in reduction of tissue viral load in severely DENV-infected AG129 mouse model

**Figure 4:**
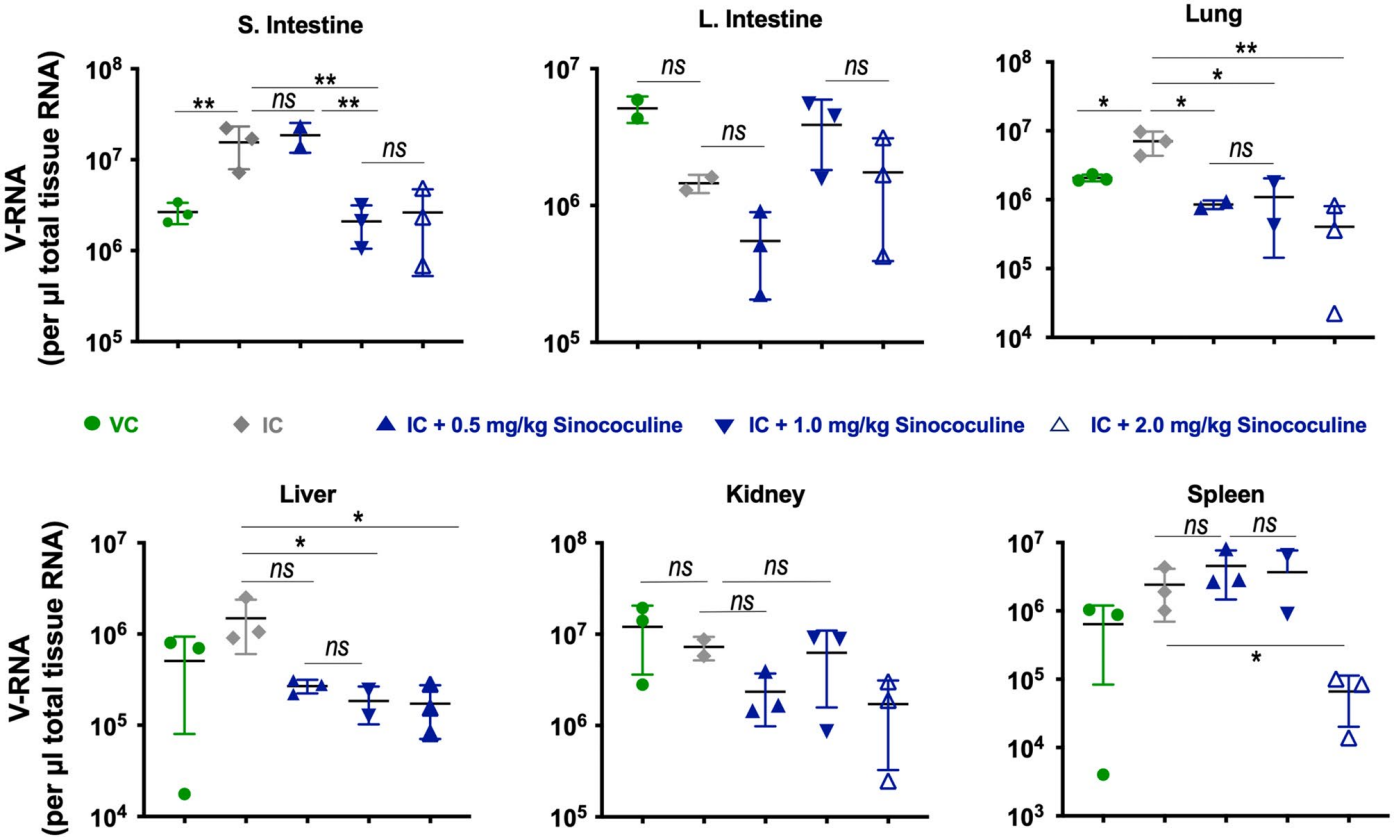
Sinococuline effectively reduces dengue virus load in several organs of severely DENV-infected AG129 mice. Group of AG129 (n = 3) inoculated with IC and treated with different doses of Sinococuline (i.p), viz., 0.5 mg/kg/day, 1.0 mg/kg/day and 2.0 mg/kg/day, in a BID manner for 4 days. On day-4 (post completion of dosing), mice were euthanised by CO_2_ inhalation followed by cervical dislocation, and extensively perfused with 1× PBS. The vital organs were collected (50 mg each organ) in 500 µl of RNA stabilising solution. Virus load in different organs (small intestine, large intestine, lung, liver, kidney and spleen) were estimated by SYBR green-based RT-qPCR. Data were normalised from the only Sinococuline control group (uninfected mice who received highest dose of Sinococuline i.e., 2.0 mg/kg/day; BID) which was considered as the baseline. */** indicates statistically significant and *ns*: not significant across all the panels while data were analysed by using Two-way ANOVA with GraphPad Prism software.

Onset of severe dengue (DHF/DSS) in secondary DENV-infection comes after 3–6 days of disease illness, where patients drop their fever below 38 °C and associated severe dengue symptoms manifests. The severe dengue disease symptoms involve plasma leaking, abdominal fluid accumulation, respiratory distress and several vital organ impairments^34,39^. This is because of the elevation of cytokines response (discussed in next section) eventually resulting in the enhanced virus replication in several vital organs. Hence, estimation of tissue viral load could be a better correlate of protection than serum viremia in DENV infection, as reported elsewhere^34^. Here, our aim was to evaluate the effect of Sinococuline treatment on the reduction of viral load in several organs of severely DENV-infected AG129 mice. Groups (n = 3) of IC inoculated AG129 mice were treated with different doses of Sinococuline, 0.5 mg/kg/day, 1.0 mg/kg/day and 2.0 mg/kg/day for four days in BID routine (described in above section). Post 96 h of IC inoculation (or from beginning of Sinococuline treatment), mice were euthanised, perfused and 50 mg (wet weight) of each tissue were collected in RNALater (500 µl). Tissues were homogenised, fractionated and 100 µl of homogenate supernatants were utilised for determination of tissue viral load by SYBR Green-based real time RT-PCR (Fig. 4) Strikingly, the viral replication was inhibited in lungs and liver as viral load in these tissues dropped significantly even with the lowest concentration of Sinococuline treatment (0.5 mg/kg/day), whereas, viral copies in small intestine were inhibited significantly by both 1.0 mg/kg/day and 2.0 mg/kg/day of Sinococuline dosing (*p* = 0.032), when compared with untreated group. However, in spleen, the viral copies were inhibited significantly only with the highest concentration of Sinococuline treatment (*p* = 0.040). Notably, the effect of Sinococuline treatment on lung tissue was the maximum. In conclusion, 2.0 mg/kg/day; BID treatment of Sinococuline inhibits virus load in almost all the organs of severely DENV-infected mice.

To estimate the Sinococuline treatment effect on liver toxicity, liver tissue was collected from each mouse utilised in Fig. 4 and fixed in natural buffered formalin solution. The liver pathology was performed from the fixed tissue for the estimation of any toxicity. The result of liver pathologies indicated that there was no adverse effect of Sinococuline treatment on liver when compared with IC inoculated AG129 mice (Supplementary Fig. 2).

### Sinococuline treatment reduces pro-inflammatory cytokines in severely infected AG129 mouse model

**Figure 5:**
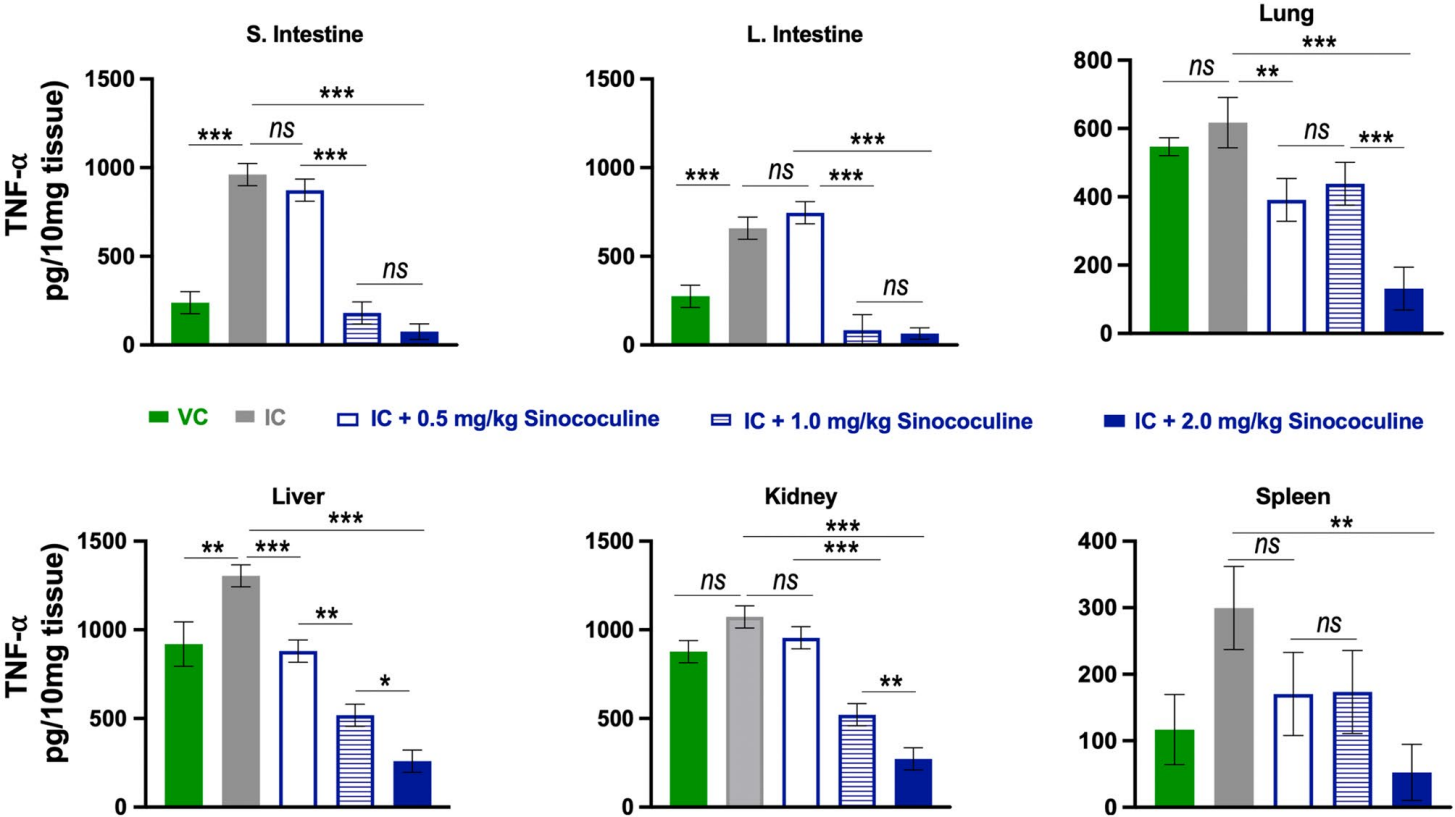
Sinococuline reduces TNF-⍺, a proinflammatory cytokine, in several organs of severely DENV-infected AG129 mice. The IC inoculated AG129 mice (n = 3) were treated with Sinococuline doses corresponding to each of the experimental groups described in Fig. 4. In parallel the mice were euthanised on day 4 post-infection, perfused and different organs were harvested for the estimation of TNF-⍺ using mouse TNF-⍺ ELISA kit. The TNF-⍺ levels in each tissue were calculated in pg/10 mg of tissue and graph were plotted by using GraphPad prism. The only Sinococuline (2.0 mg/kg/day) administered group was included and treated similarly to normalise the data across all the panels. The difference of TNF-⍺ levels between two infected or treated groups were analysed by using Two-way ANOVA with Bonferroni’s multiple comparison test; p ≤ 0.05 were considered as significant (*/**) and *p* values ≤ 0.001 were considered as very significant (***).

**Figure 6:**
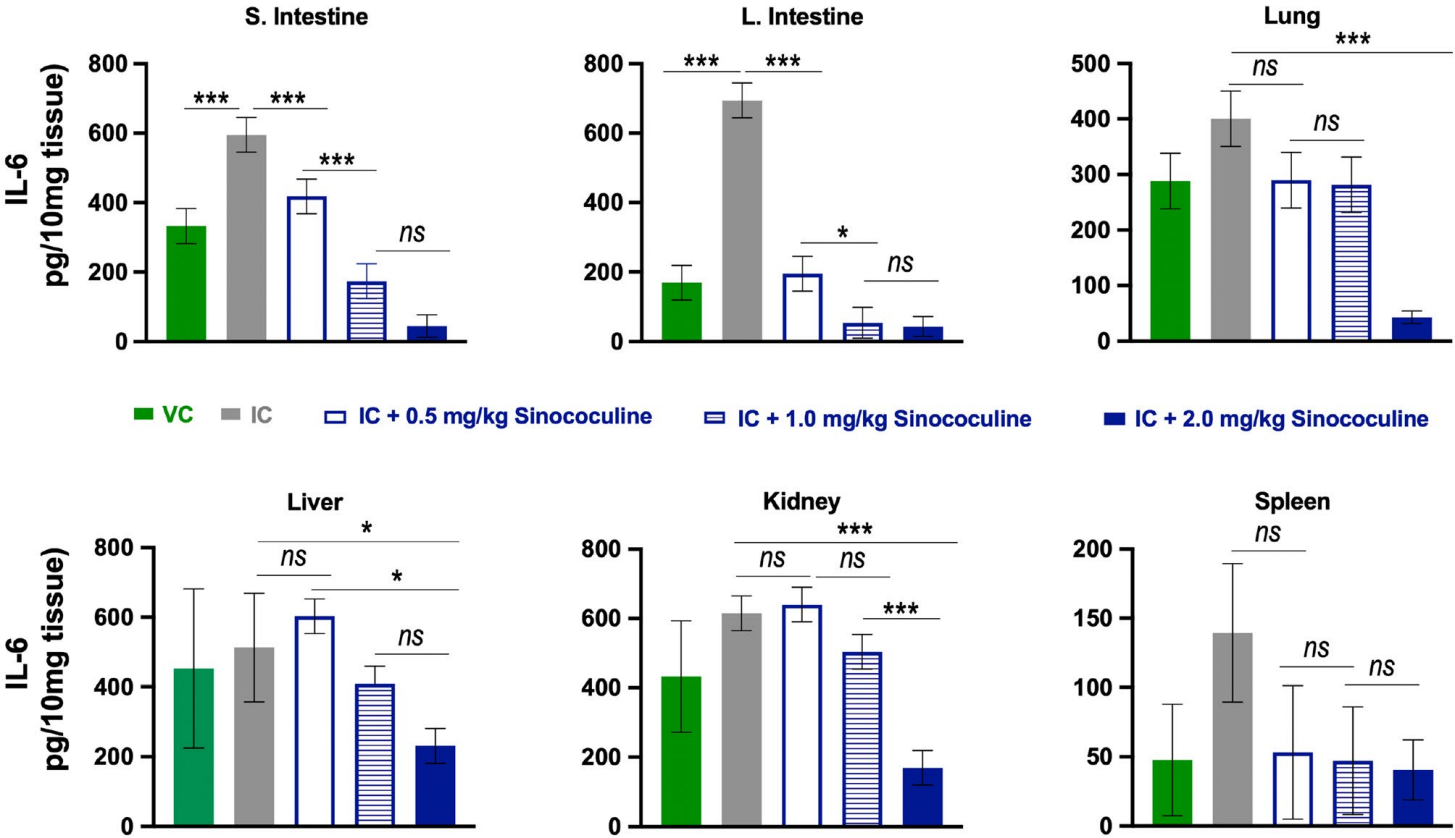
Sinococuline reduces IL-6, a proinflammatory cytokine, in different organs of severely DENV-infected AG129 mice. The 100 µl of tissue homogenate supernatants from the corresponding experimental groups described in Fig. 5, were utilized for the evaluation of IL-6 by using mouse IL-6 ELISA kit. The assay was performed as per manufacturer’s instructions, levels of IL-6 were calculated and graphs were plotted through GraphPad prism. The statistical levels of difference of IL-6 between the groups were analyzed by Two-way ANOVA with Bonferroni’s multiple comparison test, where *ns*: not significant (*p* > 0.05), ***very significant (*p* < 0.001), *significant (*p* ≤ 0.02).

DENV primarily infects mononuclear cells thus leading to major secretion of pro-inflammatory cytokines, Tumour Necrosis factor-alpha (TNF-α) and interleukin-6 (IL-6). Eventually elevated levels of inflammatory cytokines (especially TNF-α and IL-6) potentiate cascade of events that culminate in vascular permeability and haemorrhage, which is the leading cause of severe dengue illness. To test if Sinococuline had anti-inflammatory effect, we estimated the secretion levels of cytokines (Figs. 5, 6) in the IC inoculated and Sinococuline treated groups. 50 mg of each tissue from the Sinococuline treated and non-treated AG129 mice (described in above section) were homogenised in 500 µl of 1× PBS and subjected to the estimation of TNF-α (Fig. 5) and IL-6 (Fig. 6). Interestingly, Sinococuline treatment inhibited the secretion of cytokines in a dose-dependent manner in several tissues (small intestine, large intestine, liver and kidney). The expression of cytokines was dropped very significantly with the highest dose of Sinococuline treatment (2.0 mg/kg/day) in all the assayed tissues of the infected mice.

In conclusion, Sinococuline treatment effectively protects severely DENV-infected AG129 mice, as it has the potency to inhibit the viral load, inflammatory cytokines and vascular permeability, which is the hallmark of severe dengue disease.

## Discussion

With an estimated global toll of ~ 400 million annual dengue infections^13^ and a doubling of the infection rate each decade^40^, dengue has become a serious global health concern^6^. Thus, there is an urgent need for a broad-use vaccine or drug to prevent, treat and impede the spread of dengue. Several anti-viral drugs have been tested clinically, but none of the drugs succeeded to steer past the efficacy trials^19^.

Our group has been working on the exploration of herbal medicines to find a phytopharmaceutical-based cure for dengue. The classical medicinal literature worldwide reports the use of several plants in dengue like febrile illnesses. We followed Indian medicinal literature, Ayurveda, as a reference for finding a solution to our anti-dengue quest. In this endeavour, we previously identified aerial methanolic extract of *Cissampelos pareira* which had pan anti-dengue activity in vitro and in vivo^31^. We further identified the aerial methanolic extract of *Cocculus hirsutus* to be more potent than that of *C. pareira*^29^. Efforts were made to intensify anti-dengue potency of *C. hirsutus* by exploring different extraction solvents and plant parts, which led to the establishment of aqueous extract of stem of *C. hirsutus* (AQCH) to be the most efficacious^29^ in vitro and in vivo. AQCH effectively protected severely DENV-infected AG129 mice, when treated with 25 mg/kg body weight; QID for 5 days. Moreover, AQCH treatment reduced serum and tissue viral load and prevented intestinal blood vascular leakage significantly^29^. Additionally, five chemical markers- Sinococuline, Magnoflorine, 20-hydroxyecdysone, Makisterone-A and Coniferyl alcohol were identified through HPLC analysis. Out of these chemical compounds, only Sinococuline exhibited anti-DENV inhibitory property when evaluated in vitro. Furthermore, the AQCH phytopharmaceutical substance was made and evaluated clinically which was found to be very safe for human use^41^.

Concerns of limited availability of *C. hirsutus* of appropriate age, the need to cultivate it on large scale for commercial use of AQCH and the sheer bulk of operation required in collection and preparation of AQCH, warranted the need to evaluate bioactive ingredient of AQCH, which could be synthesized at industrial scale. This report highlighted the protective efficacy of the bioactive ingredient of AQCH, Sinococuline, in severely DENV-infected AG129 mouse model. The AG129 (IFN-α/β and -γ receptors deficient) mice have been used widely as it support robust levels of DENV infection and its replication^42^. Also, AG129 mice model recapitulate the mild dengue infection into a severe dengue disease by transfer of sub-protective levels of anti-DENV cross-reactive antibodies and mimics variety of dengue severe disease symptoms, including serum viremia, cytokines storm, elevated haematocrit, low platelet counts, vascular leak and intestinal haemorrhage^43,44^. Thus we explored AG129 mouse model to evaluate protective efficacy of Sinococuline. Our first quest was to identify a suitable route of Sinococuline administration, which could improve the protective efficacy in vivo. For this, severe DENV-infection in AG129 mice was established by inoculating with IC of DENV-2 S221 made with pan-DENV cross-reactive monoclonal antibody, 4G2, as reported previously^8,29^. Thereafter, groups of infected mice were treated with 10 mg/kg/day, for five days in BID and QID dosing regimen via intraperitoneal (i.p.) injection and oral gavage, respectively (Fig. 2a). Notably, the i.p. injection with 10 mg/kg/day; BID of Sinococuline protected severely DENV-infected AG129 mice (Fig. 2b) without any significant symptom score (Fig. 2c, empty square and supplementary media file) and body weight change (Fig. 2d). The IP injection of Sinococuline not only protected the infected mice but also prevented the vascular leakage compared to the orally treated mice group (Fig. 2e). The vascular leakage is considered as hallmark symptom of severe dengue illness.

After establishing the suitable route of administration and dosing regimen, we evaluated the optimum dose of Sinococuline, which could confer complete protection of severely DENV-infected AG129 mice without any severe symptoms score. We selected different doses of Sinococuline (0.5 mg/kg/day, 1.0 mg/kg/day and 2.0 mg/kg/day) and treated the IC inoculated AG129 mice for five days in a BID regimen (Fig. 3). Interestingly, we found that the 1.0 mg/kg/day and 2.0 mg/kg/day of Sinococuline treatment reduced the serum viremia significantly (*p* = 0.0013), when it was observed on day 4 post-infection. Although, the reduction of serum viremia by 1.0 mg/kg/day in comparison to 2.0 mg/kg/day of Sinococuline treatment was statistically insignificant (*p* > 0.05), 1.0 mg/kg/day Sinococuline failed to confer full protection and only ~ 40% mice survived over entire period of experiment (Fig. 3c). Whereas, survival rates of 1.0 mg/kg/day group was not significantly different with 0.5 mg/kg/day, it varied significantly with respect to their morbidity scores.

Next, we wanted to know whether the protective dose of Sinococuline has the potency to reduce the tissue viral load in different vital organs, as the virus is sequestered in tissues in advanced phase of DENV infection leading to increase in tissue viral load and elevated the proinflammatory cytokines. The subset of mice group (n = 3) utilised in Fig. 3, were euthanised on day 4, perfused, several organs were collected and tissue viral load was estimated by performing the RT-qPCR using universal DENV primers. Notably, mice treated with 1.0 mg/kg/day of Sinococuline reduced virus level in small intestine, lung and liver significantly (p < 0.01, Fig. 4), when compared with non-treated group (group of mice inoculated with IC but not treated with Sinococuline). However, the maximum dose of Sinococuline (2.0 mg/kg/day) effectively reduced the viral load in almost all the assayed organs. Surprisingly, all three selected doses of Sinococuline significantly decreased the viral load in lung tissue in all the three independent experiments.

The elevation of pro-inflammatory cytokines especially TNF-⍺ and IL-6 are a major factor to cause the severe dengue disease, as these cytokines attract and facilitate monocytes infiltration at the site of DENV deposition or infection. The DENV bound with cross-reactive antibodies (immune complex) takes this opportunity to be internalised via Fc receptor and cause intrinsic ADE which culminates into severe dengue illness. To evaluate the effect of Sinococuline in the reduction of pro-inflammatory cytokines in IC inoculated mice, tissues were collected from a subset of mice (n = 3) group utilised in Fig. 4, homogenised and subjected for TNF-⍺ and IL-6 estimation by using the commercially available kits. Interestingly, the mice treated with 1.0 mg/kg/day or 2.0 mg/kg/day of Sinococuline were able to reduce both TNF-⍺ (Fig. 5) and IL-6 (Fig. 6) significantly (*p* < 0.001) in all the assayed vital organs (Small intestine, large intestine, Lung, liver, kindly and spleen). Nonetheless, the highest concentration of Sinococuline (2.0 mg/kg/day) treated mice group observed statistically higher significant reduction in TNF-⍺ in lung, liver, kidney and spleen tissues. However, similar effect was observed in the case of IL-6 reduction only in Kidney and lung tissues (Fig. 6). It could be because of better bioavailability of Sinococuline in lungs than other tissues, but exact cause is unknown.

To evaluate any adverse effect of Sinococuline treatment on liver tissue, the liver function test was performed by the estimation of essential liver maker enzymes circulating in the blood i.e., SGOT and SGPT. The SGPT and SGOT results indicated that Sinococuline did not interfere with the function of liver cells and levels of SGPT and SGOT were not altered when compared with the non-treated group. Instead, the highest dose of Sinococuline (2.0 mg/kg/day) significantly reduced the elevated level of SGOT, compared with IC-inoculated AG129 mice who did not receive any dose of Sinococuline (Supplementary Fig. 1). However, a similar effect was not observed with SGPT levels. The Sinococuline treatment did not cause any adverse effect and was found to be safe for treatment against DENV infection in mice, as observed through liver pathology (Supplementary Fig. 2).

In conclusion, Sinococuline demonstrated its potential to control severe DENV infections in vivo. Sinococuline could be explored further as an anti-dengue compound through elaborate pre-clinical and clinical development to address the urgent need for a dengue anti-viral.

## Materials and methods

### Materials

The WHO referenced DENV strains DENV-1 (WP 74), DENV-2 (S16803), DENV-3 (CH53489), and DENV-4 (TVP-360) were used in Flow-cytometry based viral inhibitory property of Sinococuline. And, the DENV-2 strain S166803 was utilised for DENV-2 antigen (NS1) release assay as described previously^31^. DENV-2 strain S221 used in all animal related experiments was obtained from Global Vaccines Inc., NC, USA. These viruses (all four serotype of DENV and DENV-2 S221) were propagated in C6/36 cells (ATCC-CRL 1660) cells to prepare stock which were titrated on Vero cells (ATCC-CRL-1586) using a Flow-cytometry based virus infection assay^45^. Titres of the DENV stocks were expressed as FACS infectious units (FIU)/ml, as described^46,47^. AG129 mice were purchased from B&K Universal, UK, and bred in-house at ICGEB, New Delhi, for all the in vivo experiments. The monoclonal anti-flavivirus antibody D1-4G2-4–15 (4G2 mAbs) was produced in-house by using hybridoma, procured from ATCC (ATCC HB-112). The MTT reagent [3-(4, 5-dimethylthiazolyl-2)-2,5-diphenyltetrazolium bromide] was procured from Sigma Aldrich, USA (Cat. # M5655) and used for in vitro evaluation of cytotoxicity of Sinococuline. Batches of Sinococuline was purified from AQCH (Aqueous Extract of *Cocculus hirsutus*) at Sun Pharmaceutical Industries Ltd, Gurugram (Supplementary table 1), as described earlier^29^ and the purified Sinococuline [Batch no. SP(I-663)084; Purity: 89.8%] was used for all in vitro and in vivo experiments. The levels of TNF-α and IL-6 were determined by using mouse TNF-α (Invitrogen: cat# KMC3011) and IL-6 (Invitrogen: cat# KMC0061) kits.

### Evaluation of cytotoxicity of Sinococuline

The cell cytotoxicity of Sinococuline was evaluated through MTT assay. The Vero cells were seeded in 96-well plate (2.0 × 10^4^ cells/well) one day prior to the exposure of Sinococuline. A wide range of Sinococuline (0–40 µg/ml) was added in a volume of 200 µl of media (1× DMEM + 2.0% ΔFBS) and incubated at 37 °C, in a humidified condition for another 48 h. Post-incubation, 20 µl of MTT reagent (5 mg/ml) was mixed in the overlay media and incubated further for 2 h in above mentioned conditions. Post-incubation, overlay media mixed with MTT reagent was aspirated and 100 µl of DMSO was added and allowed to dissolve the formed formazan crystal. After complete dissolution of crystals, absorbance at 570 nm was taken and percentage of half-maximal cytotoxic concentration (% CC_50_ value) for Sinococuline was calculated with respect to the untreated cells (positive control) which observed 0% cytotoxicity and 100% cell viability. Selective index (SI) of Sinococuline was defined by dividing of CC_50_ with IC_50_ (SI = CC_50_/IC_50_) and in vivo evaluation of Sinococuline was carried out within the SI limit.

### DENV antigen release assay

DENV antigen release assay was done using Vero cells as described earlier with minor modifications. The DENV infected cells secrete viral non-structural protein-1 (NS1) which can be scored in the culture medium. In brief, seeded Vero cells (2.0 × 10^4^ cells) were exposed with 0.01 MOI of DENV-2 for 2 h at 37 °C, 10% CO_2_ in humidified incubator. Post-infection, overlay media was aspirated, and media (1× DMEM + 2.0% ΔFBS) supplemented with different concentration of Sinococuline (0.5 µg/ml, 1.0 µg/ml, 2.0 µg/ml and 5.0 µg/ml) in a total volume of 200 µl, was added and incubated for another six days. An aliquot was collected every 24 h intervals for six days and NS1 capture ELISA was carried out by using Dengue NS1 Ag Microlisa kit (J Mitra & Co. cat# IR031096) as per manufacturer’s instructions. In brief, the 100 µl of diluted aliquoted culture supernatant (1:250) was added onto the anti-Dengue NS1 antibodies precoated microwells and incubated at room temperature (RT) for 1 h. Thereafter, microwells were washed 5× with the provided 1× washing buffer and monoclonal anti-dengue NS1 antibodies linked to Horseradish peroxidase was added and incubated further at RT for 1 h. Wells were washed 5× with washing buffer and enzyme substrate was added and incubated at 37 °C for 30 min. Reaction was stopped with 1 N H_2_SO_4_ and absorbance at 450 nm was taken and graph was plotted.

### Preparation of immune complex of DENV-2 S221 to establish severe DENV infection in AG129 mouse model

In order to establish severe DENV-infection in AG129 mouse (an interferon type I and II receptor-deficient mice) model, we made 100% neutralised immune complex (IC) of DENV-2 strain S221 by mixing of 4G2 mAb (10 µg) with 2.0 × 10^4^ FIU of DENV-2 S221 in vitro*,* as described elsewhere^20,45^.

### Determination of appropriate route of Sinococuline administration in AG129 mice

To obtain maximum effect of Sinococuline, we adopted two routes of Sinococuline administration in severely DENV-infected AG129 mouse model. Groups of IC inoculated AG129 mice (n = 9) were either fed orally or intraperitoneally (i.p) injected with 10 mg/kg/day of Sinococuline (corresponding to the effective concentration of active ingredient, Sinococuline in AQCH)^29^ in two different dosing regimens, twice a day (BID) and four-times a day (QID), respectively for five days. All the treated mice were monitored for their survival, morbidity and body weight change for 15 days post-infection. The morbidity score was measured based on a 5-point scale system, mild ruffled fur: 0.5; ruffled fur: 1.0; compromised eyes: 1.5; hunched back along with compromised eyes: 2.0; loose stools: 2.0; limited movement: 3.0; no movement on stimulus/hind leg paralysis: 3.5; euthanized if the cumulative score was 4: 4.0; observed dead during experiment: 5.0, as reported elsewhere^29,45^. Only Sinococuline group (orally fed with 10 mg/kg/day; QID) was included as experimental control and treated similarly, however, the IC group was included as a vehicle/placebo control where animals were infected with the IC like test groups but were administered with sterile 1× PBS instead of Sinococuline treatment. Post 15-days, Kaplan–Meier survival graph was plotted and statistical differences between two doing route were analysed by Long-rank (Mantel-Cox) test, p > 0.05 considered as not significant.

Moreover, subset (n = 3) of above-mentioned experimental groups, were euthanised on day-4, perfused with 1x PBS (50 ml) and luminal content was further flushed with 1× PBS (25 ml) in order to remove any luminal content, and visualised qualitatively for intestinal vascular leakage.

### in vivo evaluation of Sinococuline in severely DENV-infected AG129 mouse model

Severe DENV-infection was established in AG129 mice by intravenously (i.v.; retroorbital) injecting them with in vitro made IC of DENV-2 S221 virus (10 µg 4G2 mAb + 2.0 × 10^4^ FIU DENV-2 S221 per mouse) in a volume of 50 µl, as described^29^.Group of AG129 mice (n = 9) were injected *i.v.* with IC, thereafter, mice were treated with different doses of Sinococuline viz*.,* 0.5 mg/kg/day, 1.0 mg/kg/day and 2.0 mg/kg/day in a total volume of 100 µl (made in sterile 1× PBS), twice a day (BID) dosing regimen for five days. All the animals were monitored for 15 days post-infection and the level of protection conferred by Sinococuline were observed through mortality and morbidity score of the animals. The morbidity scores were assessed by using 5-point scale as described in above section. Controls, only sublethal dose of virus (V: 2.0 × 10^4^ FIU), IC and only Sinococuline (sham groups) were included and treated similarly. Fifteen days post-infection, the level of protection was evaluated by plotting the Kaplan–Meier survival graph and % survival was scored. These experiments were done three times independently and one of the data set is represented in this manuscript.

### Estimation of Serum viremia and cellular viral load in Sinococuline treated AG129 mice

IC inoculated and Sinococuline treated mice as described in above section, were bled on day-4 for the estimation of serum viremia as reported previously^29^. 50 µl of serum was utilised per mouse for the isolation of viral RNA using QiAamp Viral RNA Mini Kit (Qiagen, cat# 52904) as per manufacturer instructions. cDNA was made from the isolated genomic RNA by using iScriptTM Select cDNA Synthesis Kit (BioRad, cat# 1708897) along with DENV-2 specific reverse primer (5′CGCGTTTCAGCATATTGAAAG3′), subsequently, cDNA was utilised for qPCR using DENV-2 specific forward and reverse primers (Forward primer: 5′-AGTTGTTAGTCTACGTGGACCGA-3′; reverse primer: 5′-CGCGTTTCAGCATATTGAAAG-3′) in combination with iTaq Universal SYBR Green Super Mix (BioRad, cat# 1725124) in a CPX96 Real-Time PCR system (BioRad). Sham group were treated similarly and considered as base line while plotting the graph for the RT-qPCR data. The statistical differences between two doses were analysed by Two-way ANOVA with Tukey’s Multiple comparison test and p < 0.05 were considered as significant.

For cellular viral load, subset of experimental mice (n = 3) described in the section above (in vivo evaluation of Sinococuline), were euthanised, perfused extensively with sterile 1x PBS (50 ml) and 50 mg of each tissue (small intestine, large intestine, lung, liver, kidney, spleen) were collected in RNA stabilising reagent (RNALater: Invitrogen, cat# AM7020), homogenised by using Polytron homogeniser and total tissue RNA was purified using RNeasy Plus Mini Kit (Qiagen, cat# 74134) as per manufacturer’s protocol. The isolated total RNA was utilised for doing RT-qPCR by using SuperScript III Platinum One step Quantitative RT-PCR System with ROX (Invitrogen, cat# 11745-100) as per manufacturer’s protocol along with forward (5′-CATATTGACGCTGGGAAAGA-3′) and reverse (5′-AGAACCTGTTGATTCAAC-3′) primers for DENV quantification as described earlier^45^.

### Estimation of liver enzymes in severely infected AG129 mice

Liver enzymes, namely, SGOT (Serum glutamate oxaloacetate transaminase) and SGPT (Serum Glutamic Pyruvic Transaminase) were quantified in Sinococuline treated AG129 mice using standard commercial assay kits (SGOT, Cat#1102200075 and SGPT, Cat# 1102210075, Coral clinical system, Tulip Group, India) and experiment was performed as per manufacturer’s instructions.

### Estimation of cytokines response in Sinococuline treated AG129 mice

Groups of AG129 (n = 3) were inoculated with IC, in order to establish severe DENV-infection. The infected mice were treated similarly as above with three different doses of Sinococuline (0.5 mg/kg/day, 1.0 mg/kg/day and 2.0 mg/kg/day; BID) for 4 days. On day 4, (post dosing), animals were euthanised by CO_2_ inhalation and death was confirmed by cervical dislocation. Thereafter, mice were perfused with 50 ml of sterile 1× PBS and collected several vital organs (Small intestine, large intestine, lung, liver, kidney, spleen). 50 mg (wet weight) of each tissue were homogenised in 500 µl of 1× PBS and and clarified by centrifugation at 5000 rpm for 10 min. 100 µl of each organ’s clarified supernatant were utilised for the estimation of Tumour necrosis factor-a (TNF-α) and interleukin-6 (IL-6) by using commercial ELISA kits as per manufacturer’s protocol. The provided biotinylated TNF-α and IL-6 standards were considered as reference for the calculation of tissue’s cytokines. The levels of cytokines in only Sinococuline mice group (sham group; 2.0 mg/kg/day; BID) was treated similarly and considered as base line for the normalization of the data.

### Statistical analyses

GraphPad prism software (version 9.3.) was used for all the statistical calculations. Probability (*p*) value < 0.05 was considered as significant and *p* < 0.0001 observed as very significant.

### Ethical approval

The AG129 mice involved in this study was approved (ICGEB/IAEC/30012021/RGP-2) by the Institutional Animal Ethics Committee (IAEC) of International Centre for Genetic Engineering and Biotechnology (ICGEB), New Delhi and all the methods were performed in accordance with Experimental Animals (CPCSEA) guidelines of Government of India. Also, this manuscript confirming the study is reported in accordance with ARRIVE guidelines.

## Supplementary Information

Supplementary Video 1.

Supplementary Information 1.

## Data Availability

All data generated or analysed during this study are included in this article (and its Supplementary Information file).
