# Supplementary material for: Sinococuline, a bioactive compound of Cocculus hirsutus has potent anti-dengue activity
Source: Sci Rep. 2023 Jan 19;13:1026. doi: 10.1038/s41598-023-27927-3 (PMC9852271; doi:10.1038/s41598-023-27927-3)
Supplement: Supplementary file 2 — Supplementary Information 1. [file 41598_2023_27927_MOESM2_ESM.pdf]

'Sinococuline, a bioactive compound of *Cocculus hirsutus* has potent anti-dengue activity'

Rahul Shukla[1,2], Richa Ahuja[1,3], Hemalatha Beesetti[3a], Amit Garg[1], Charu Aggarwal[1], Shivam Chaturvedi[1], Kaushal Nayyar[3], Upasana Arora[3], Altaf A. Lal[3] and Navin Khanna[1,4*]

1Translational Health, Molecular Medicine Division, International Centre for Genetic Engineering & Biotechnology, New Delhi, India

2Division of Virus Research and Therapeutics, CSIR-Central Drug Research Institute, Lucknow, India

3Sun Pharmaceutical Industries Limited, Gurugram, India,

4Translational Health Science & Technology Institute, NCR Biotech Science Cluster, Faridabad, INDIA

aPresent Address: Virology Division, Foundation for Neglected Disease Research, 20A, KIADB Industrial Area Veerapura, Doddaballapur, Bengaluru, Karnataka 561203 (HB).

Supplementary Materials

Supplementary Table 1: *Batches of Sinococuline purified from three consecutive batches of AQCH.*

Sl. No	Sinococuline batch no.	Purity (%)
1.	*SP(I-663)084*	*89.8*
2.	*SP(J-191)021*	*89.0*
3.	*SP(I-663)056*	*72.8*

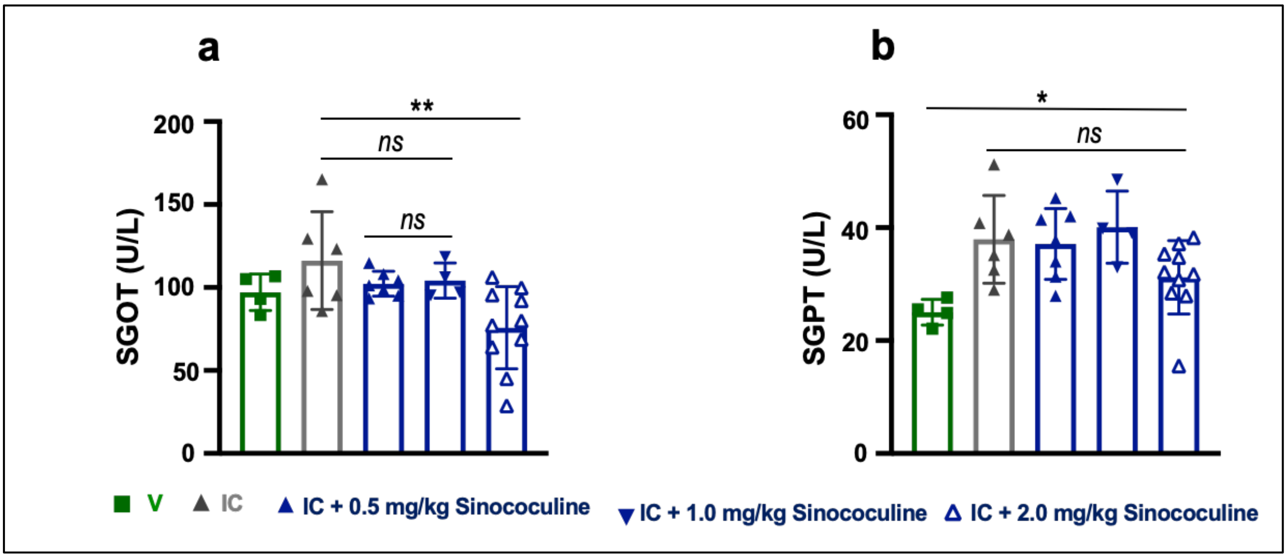

Supplementary Figure 1: Profile of Liver function of IC inoculated and Sinococuline treated AG129 mice (a) *SGOT (Serum glutamic-oxaloacetic transaminase), and* ***(b)*** *SGPT (Serum glutamic pyruvic transaminase) enzymes levels were estimated in IC inoculated AG129 mice (n=5 – 10) who were treated with three concentration of Sinococuline viz., 0.5 mg/kg, 1.0 mg/kg and 2.0 mg/kg body weight. Blood was collected from each mouse of the groups (treated and non—treated group) at day 4 post infection and subjected for the evaluation of liver function by using standard commercial assay kits of SGOT and SGPT. The IC + 2.0 mg/kg Sinococuline treated mice were showed significant reduction in SGOT (p=0.0059), however, statistically significant differences between individual groups were determined by using One-way ANOVA with Tukey's multiple comparisons test. The **/* detnotes significant difference between the group (**p=0.0059; *p=0.02) and ns indicates not significant difference.*

a

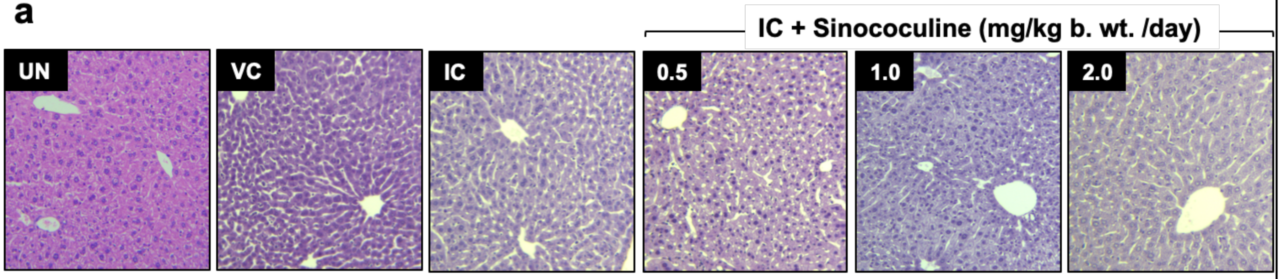

b

Parameters	UN	VC	IC	IC + Sinococuline (mg/kg b.wt./day)		
				0.5	1.0	2.0
**Degeneration**	Absent	Minimal	Mild	Mild	Absent	Absent
**Congestion/ Haemorrhages**	Absent	Absent	Absent	Absent	Absent	Absent
**Necrosis**	Absent	Absent	Absent	Absent	Absent	Absent
**Fatty changes/cytoplasmic vacoulation**	mild	Mild	Mild	Mild	Absent	Mild
**Nuclear condensation**	Absent	Present	Absent	Absent	Present	Absent
**Hepatic cord disruption**	Absent	Marked	Mild	Mild	Mild	Absent

Supplementary Figure 2: Liver histopathology of IC inoculated and Sinococuline treated AG129 mice. *AG129 mice (n=3) were inoculated with IC, thereafter, treated with three different doses of Sinococuline (0.5 mg/kg/day, 1.0 mg/kg/day and 2.0 mg/kg/day) in BID dosing schedule for a period of 5 days. On day 4 post-infection, all mice were euthanized and perfused with 1X sterile PBS and liver tissues were collected in 10% neutral buffered formalin (1 ml) and fixed for next 24 hrs. After 24 hrs, tissues were processed for parafilm embedding. Embedded tissues were sectioned at 5.0 µm and followed for hematoxylin and eosin staining. Stained tissue sections were visualized on 10X objective as shown in panel* ***'a'*** *and photomicrographs were analyzed for any adverse effect by Sinococuline treatment in compared to controls as represented in panel* ***'b'****. Controls, uninfected (UN), VC (Virus control) and IC mice tissues were processed similarly and one of the representative photomicrographs from each assayed group is shown here.*

Supplementary media file:

The media file showing videos of AG129 mice administered with IC and treated with Sinococuline (10 mg/kg/day) either by oral gavage (QID) or IP injection (BID) and clinical symptoms were observed as shown in the movie. The mice treated by IP injection of Sinococuline (10 mg/kg/day; BID) barely developed any clinical symptoms as all mice looks healthy (placed in far-right panel of the video), however, mice treated with same dose of Sinococuline by oral gavage (QID) were developed higher level of morbidity in compared with mice who treated through IP injection of Sinococuline. and failed to confer complete protection as shown in Fig. 2b. The morbidity appeared in controls as expected as IC inoculated mice who did not received any dose of Sinococuline, were developed higher magnitude of clinical symptoms (second panel from left) and only Sinococuline (10 mg/kg/day) fed mice were observed normal as shown in the third panel of the movie.
